# Predicting extremely low body weight from 12-lead electrocardiograms using a deep neural network

**DOI:** 10.1038/s41598-024-55453-3

**Published:** 2024-02-26

**Authors:** Ken Kurisu, Tadahiro Yamazaki, Kazuhiro Yoshiuchi

**Affiliations:** Department of Stress Sciences and Psychosomatic Medicine, Graduate School of Medicine, The University of Tokyo, Tokyo, Japan

**Keywords:** Psychiatric disorders, Arrhythmias

## Abstract

Previous studies have successfully predicted overweight status by applying deep learning to 12-lead electrocardiogram (ECG); however, models for predicting underweight status remain unexplored. Here, we assessed the feasibility of deep learning in predicting extremely low body weight using 12-lead ECGs, thereby investigating the prediction rationale for highlighting the parts of ECGs that are associated with extremely low body weight. Using records of inpatients predominantly with anorexia nervosa, we trained a convolutional neural network (CNN) that inputs a 12-lead ECG and outputs a binary prediction of whether body mass index is ≤ 12.6 kg/m^2^. This threshold was identified in a previous study as the optimal cutoff point for predicting the onset of refeeding syndrome. The CNN model achieved an area under the receiver operating characteristic curve of 0.807 (95% confidence interval, 0.745–0.869) on the test dataset. The gradient-weighted class activation map showed that the model focused on QRS waves. A negative correlation with the prediction scores was observed for QRS voltage. These results suggest that deep learning is feasible for predicting extremely low body weight using 12-lead ECGs, and several ECG features, such as lower QRS voltage, may be associated with extremely low body weight in patients with anorexia nervosa.

## Introduction

Deep learning has been widely used in recent medical research, such as automatic diagnosis from clinical images^1–4^, recognition of human genes^5^, and cognitive neuroscience^6,7^. This technique also aids in electrocardiogram (ECG) pattern recognition, such as predicting demographic features^8^ and automatically identifying cardiovascular comorbidity^9–11^. Notably, several studies have employed deep learning to estimate obesity, defined as body mass index (BMI) > 25 kg/m^2^, from 12-lead ECGs, achieving moderate predictive accuracy^12,13^.

However, models predicting extremely low body weight using a cohort of low-weight individuals, such as patients with anorexia nervosa (AN), remain unexplored. Patients with AN frequently show ECG abnormalities such as QT prolongation and bradycardia^14,15^, for which guidelines and reviews recommend ECG monitoring^16–18^. These suggest the plausibility of inversely predicting extremely low body weight using ECG. Such a prediction could be useful for patients with AN who deny their disease status^19^ and falsify their weight, e.g., by hiding heavy objects in their clothes^20^, and for those in intensive care^21^, for whom standard weight measurements may be difficult to perform. Furthermore, if such a prediction is feasible, examining the rationale of the prediction could potentially lead to a deeper physiological understanding of ECG changes in patients with AN.

Thus, the present study had two objectives. The first was to assess the feasibility of using deep learning to predict extremely low body weight from 12-lead ECGs, which may be useful in specific cases, such as patients with AN attempting to falsify their weight. The second was to highlight the parts of ECGs associated with extremely low body weight by investigating the rationale of the model prediction.

## Methods

### Ethics approval

The present study was approved by the Institutional Review Board of the University of Tokyo (approval number: 3375-(10)). Due to the retrospective nature of the study using anonymized data, informed consent was obtained using an opt-out approach. This study was conducted in accordance with the ethical standards of the 1964 Helsinki Declaration and its later amendments or comparable ethical standards.

### Study participants and measurement

The present study included patients admitted to the Department of Psychosomatic Medicine at the University of Tokyo Hospital between November 2006 and June 2023. Most patients were underweight, primarily due to AN, and were hospitalized for nutritional rehabilitation. This cohort also included patients with other types of eating disorders, such as bulimia nervosa, and a small number of patients with other psychiatric and psychosomatic disorders.

During hospitalization, patients’ weights were measured weekly. Psychosomatic physicians measured the weight of patients with lightweight clothing after body checks. Patient height was recorded upon admission. In addition, 12-lead ECGs were typically recorded upon admission or during outpatient visits. The present study used ECGs measured on the nearest day within a month (31 days) of each weight measurement.

The records were randomly split into training (80%) and test (20%) datasets. To avoid data leakage during training, data splitting was performed on an individual basis; when multiple pairs of weight and ECG belonged to a single patient, resulting from repeated admissions, all pairs were allocated to the same set.

This dataset is not publicly accessible, and no relevant studies have used the same dataset.

### Preprocessing of 12-lead electrocardiogram

The model input was a 12-lead ECG waveform formatted as a one-dimensional (1D) array of 12 channels. At the University of Tokyo Hospital, the waveform was recorded at 500 Hz for 10 s, resulting in an array of 5000 values (Fukuda Denshi, Tokyo). We explored the following preprocessing methods: (1) normalizing each waveform to have a mean voltage of 0.0 mV and a variance of 1.0 mV^2^; (2) reducing ECGs from a 10-s format to shorter segments, with randomly chosen starting points, following studies using durations shorter than 10 s^9,22,23^; (3) downsampling ECGs from 500 Hz to a lower frequency (e.g., to 100 Hz by selecting one out of every five data points), in line with relevant studies using frequencies lower than 500 Hz^9,22,23^; (4) denoising using discrete wavelet transform (DWT), which involved decomposition into eight sub-bands using Daubechies-4 wavelet, setting high-frequency noise (level 1 detail coefficient) and baseline wander (level 8 approximation coefficient) to zero, and applying inverse DWT to reconstruct ECGs (details described in previous studies^22,23^); and (5) setting any one of the 12 leads to zero. We evaluated the impact of each method and the degree of downsampling on prediction accuracy in cross-validation to determine their inclusion in the final model, as described in the following sections.

### CNN model structure

A previous study revealed that the optimal BMI cutoff point for predicting the onset of refeeding syndrome, a severe complication of AN, was 12.6 kg/m^2^^24^. Thus, we developed a convolutional neural network (CNN) that inputs a 12-lead ECG waveform to output a binary prediction of whether BMI is ≤ 12.6 kg/m^2^.

**Figure 1:**
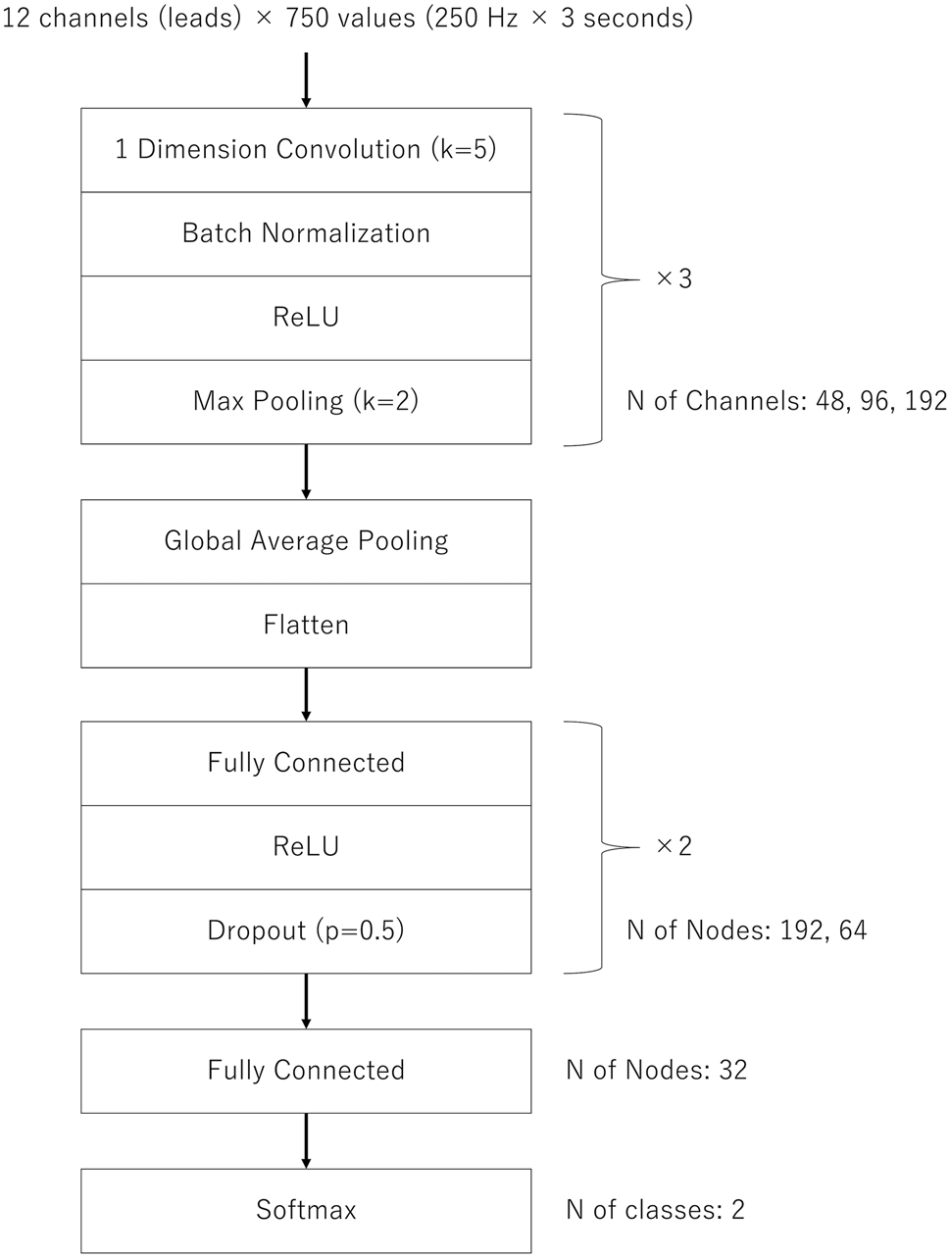
Structure of deep learning model. The network consists of three blocks of one-dimensional convolutional layers and three blocks of fully connected layers. Kernel size, pooling size, number of channels in the convolution layers, and number of nodes in fully connected layers are indicated. *ReLU* rectified linear unit.

In the present study, the CNN model was designed for processing 1D data, similar to previous research using CNNs for processing ECG data^8–12^. To the best of our knowledge, no widely used models have been designed for 1D ECG datasets, unlike models such as ResNet, which are designed for processing 2D images. Therefore, referencing a model that successfully predicts obesity from 12-lead ECGs in a previous study^12^, we prepared a network with the same structure, consisting of three blocks of 1D convolutional layers and three blocks of fully connected layers (Fig. 1).

Each convolution layer had a kernel size of 5, followed by batch normalization, rectified linear unit, and max pooling with a size of 2. The numbers of output channels in the convolution layers were 48, 96, and 192 for each block. The features extracted from the convolution blocks underwent a global average pooling layer and were flattened into an array of 192 values and then processed using fully connected blocks containing 192, 64, and 32 nodes. Finally, a softmax layer outputs the binary probability from an array of 32 values. To prevent overfitting, we added dropout layers between the fully connected blocks, as our dataset was smaller than that of the referenced study^12^.

### Model development process

AdamW with cross-entropy loss was used as the optimizer. In addition to determining whether to perform the aforementioned preprocessing methods, the batch size, number of epochs, learning rate, and weight decay were fine-tuned. These preprocessing methods and hyperparameters were selected to maximize the area under the curve (AUC) of the receiver operating characteristic (ROC) curve in fivefold cross-validation within the training dataset. Thereafter, the prediction performance was evaluated using the test dataset.

Deep learning model development was implemented using Python 3.9.18 and PyTorch 2.1.0. Statistical analyses were performed using R version 4.3.1.

### Prediction rationale interpretation

The following analyses were performed using the test dataset to evaluate the ECG features associated with model predictions. First, the final (i.e., third) 1D convolutional layer output was visualized using the gradient-weighted class activation map (Grad-CAM). This 1D heatmap was resized through interpolation and overlaid on a two-dimensional plot of the 12-lead ECGs^12^. Additionally, the correlation coefficient between the prediction score (probability of BMI ≤ 12.6 kg/m^2^) and ECG features, such as heart rate, was quantified. These ECG features were calculated from the ECG waveform independently from the model development and were not directly used as the model input.

## Results

### Dataset characteristics

**Figure 2:**
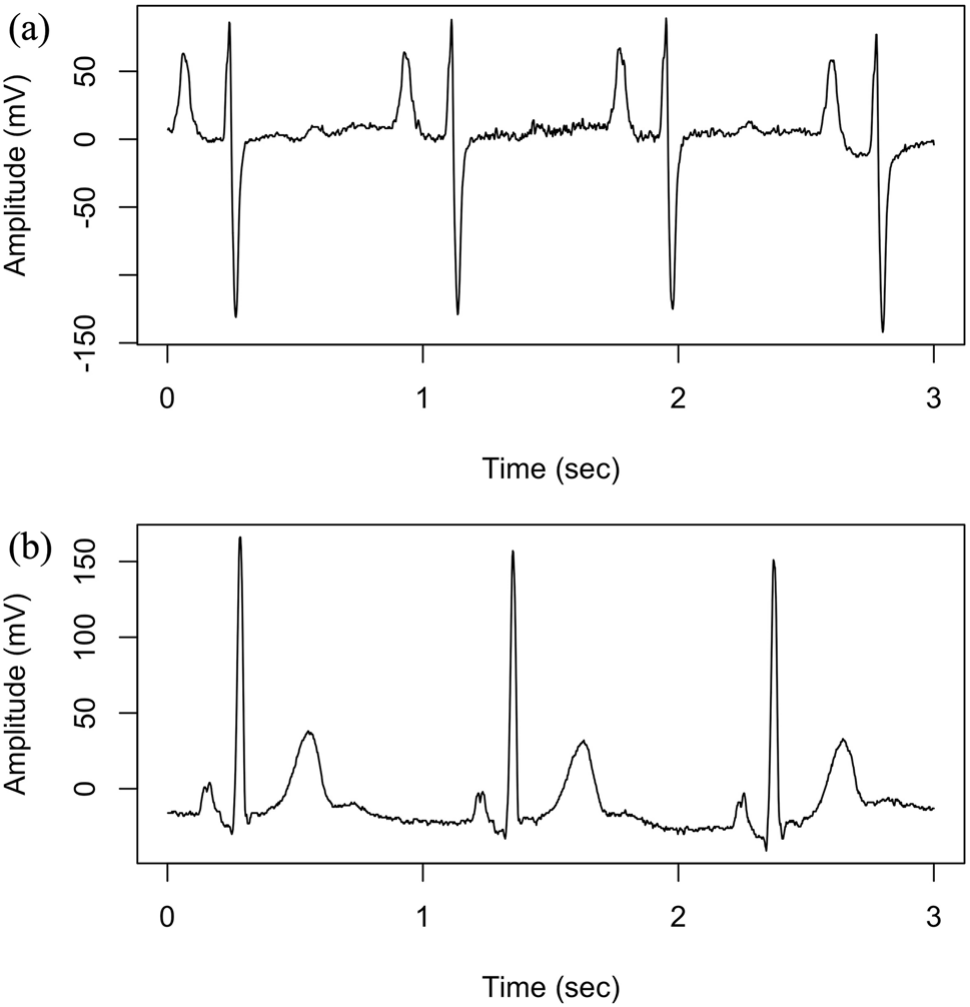
Typical examples of 12-lead ECG. Lead II wave of an individual with (**a**) BMI ≤ 12.6 kg/m^2^ and (**b**) BMI > 12.6 kg/m^2^.

A total of 888 pairs of ECG and BMI data from 391 inpatients were available. The median age (range) was 23 (12–84) years, with 857 (96.5%) of the cases being female. The median BMI (range) was 13.6 (9.2–45.0), and 316 cases (35.6%) had a BMI below the cutoff (≤ 12.6 kg/m^2^). Figure 2 shows typical examples of ECGs for an individual with a BMI ≤ 12.6 kg/m^2^ and another with a BMI > 12.6 kg/m^2^. Of these, 701 sets were allocated to the training dataset and 187 to the test dataset.

### Prediction accuracy

Among the preprocessing methods examined, normalization, shortening the duration from 10 to 3 s, and downsampling the frequency from 500 to 250 Hz contributed to improvements in AUC during fivefold cross-validation and thus were incorporated into the final model. Conversely, models using DWT and setting several leads to zero exhibited lower AUC than those without this preprocessing; therefore, these methods were not employed. After fine-tuning the hyperparameters, the average AUC in the fivefold cross-validation reached 0.787 using a batch size of 256, 25 epochs, a learning rate of 0.005, and a weight decay of 0.005. These parameters were employed for test prediction.

**Figure 3:**
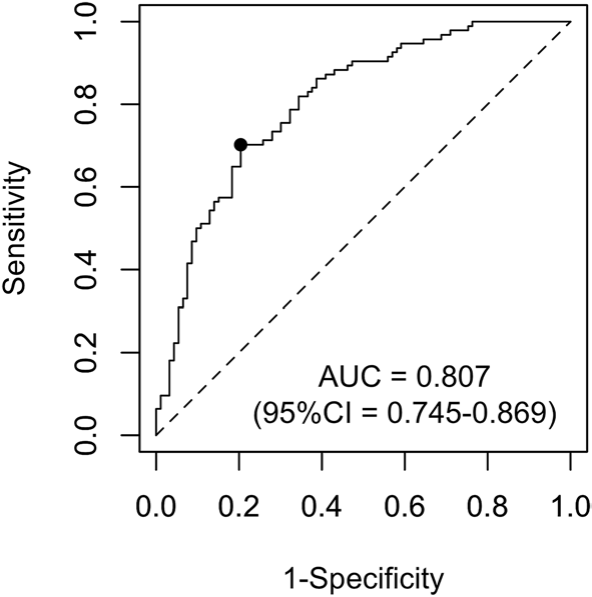
Receiver operating characteristic curve of the prediction for the test dataset. The dot indicates the cutoff point that maximizes the Youden index.

The test AUC of the final CNN model was 0.807 (95% confidence interval [CI], 0.745–0.869), achieving a sensitivity of 0.702 and a specificity of 0.796 for the Youden index maximum cutoff (see Fig. 3 for the ROC curve). Table 1 shows the confusion matrix at this optimal cutoff point. The overall accuracy was 0.749; the positive predictive value was 0.776; and the negative predictive value was 0.725.

**Table 1:** Confusion matrix of the model.

		Model prediction	
		BMI ≤ 12.6 kg/m^2^	BMI > 12.6 kg/m^2^
Label	BMI ≤ 12.6 kg/m^2^	66	28
	BMI > 12.6 kg/m^2^	19	74

### Visualization of heatmaps from Grad-CAM

**Figure 4:**
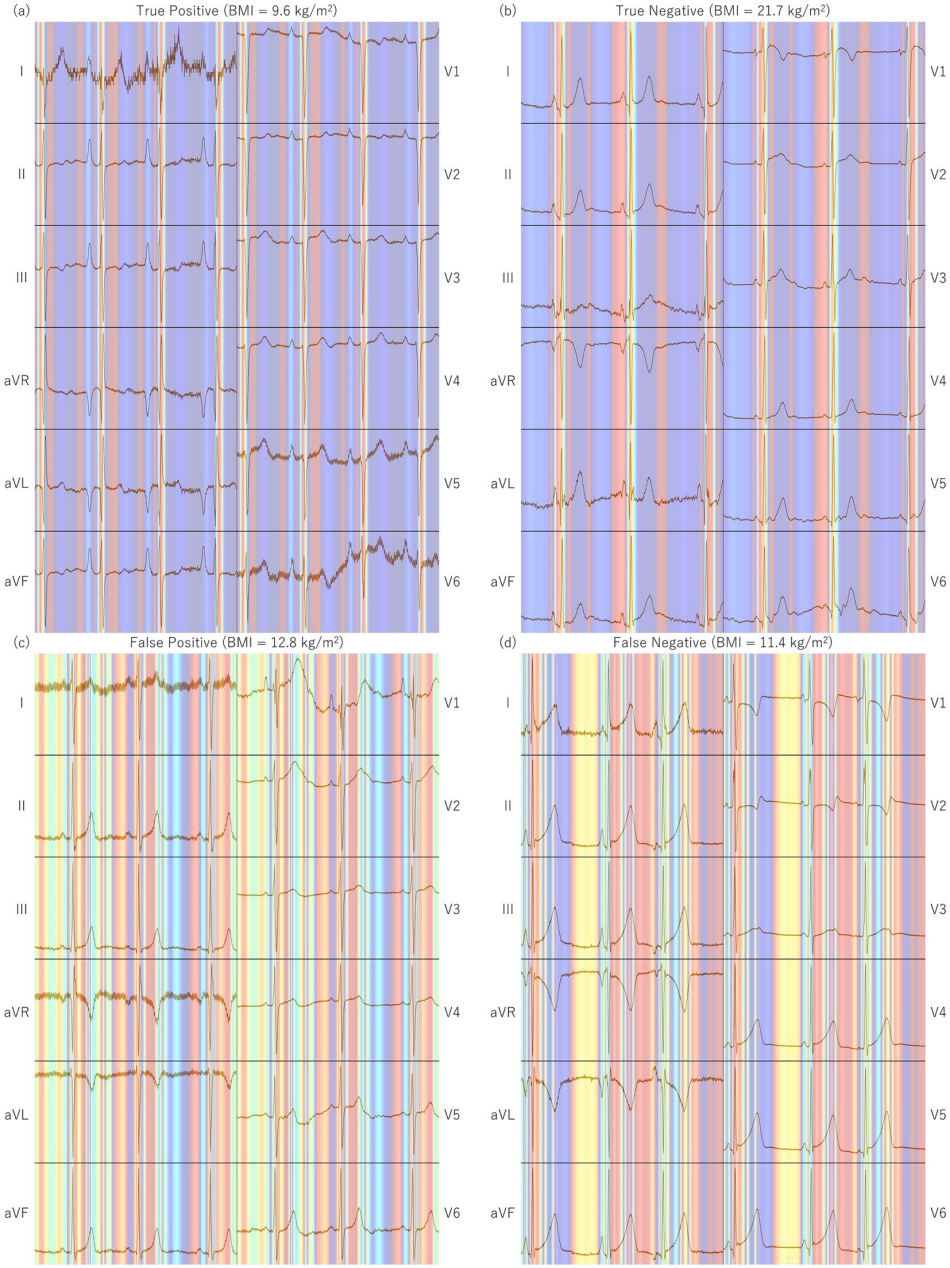
Examples of the heatmap generated by Grad-CAM. (**a**) True positive (high prediction score and BMI ≤ 12.6 kg/m^2^), (**b**) true negative (low prediction score and BMI > 12.6 kg/m^2^), (**c**) false positive (high prediction score but BMI > 12.6 kg/m^2^), and (**d**) false negative (low prediction score but BMI ≤ 12.6 kg/m^2^).

Figure 4 shows examples of heatmaps generated by Grad-CAM overlaid on the ECG plots. Areas in red-to-yellow hues greatly influence the prediction, whereas areas closer to blue have minimal impact. In both true positive (high prediction score and BMI ≤ 12.6 kg/m^2^) and true negative (low prediction score and BMI > 12.6 kg/m^2^) examples, the model appeared to primarily focus on the QRS waves. By contrast, in false positive (high prediction score but BMI > 12.6 kg/m^2^) and false negative (low prediction score but BMI ≤ 12.6 kg/m^2^) examples, no specific areas of focus were observed.

### Correlation between ECG features and model’s prediction score from waveform

Table 2 lists the descriptive statistics of the ECG features and the correlation coefficients with the prediction scores of the test dataset. The prediction scores range from 0.0 to 1.0 and approximate the likelihood of BMI ≤ 12.6 kg/m^2^. A relatively strong negative correlation was observed between the prediction scores and QRS voltage.

**Table 2:** ECG features and their correlation coefficients with prediction scores.

	Mean (SD)	Correlation coefficient with prediction score (95% CI)	P-value
Heart rate (1/min)	60 (14)	−0.001 (−0.152 to 0.135)	0.91
RR interval (ms)	1046 (234)	0.030 (−0.114 to 0.173)	0.68
PR interval (ms)	152 (22)	0.035 (−0.109 to 0.178)	0.63
QRS interval (ms)	90 (9)	−0.040 (−0182 to 0.104)	0.59
QTc interval (ms)	411 (35)	0.121 (−0.023 to 0.260)	0.098
QT dispersion (ms)	50 (16)	0.131 (−0.013 to 0.269)	0.074
P axis degrees	61 (33)	0.294 (0.157 to 0.419)	** < 0.001**
QRS axis degrees	71 (49)	−0.116 (−0.255 to 0.028)	0.11
R voltage V6 (mV)	0.73 (0.35)	−0.605 (−0.689 to −0.505)	** < 0.001**
S voltage V1 (mV)	0.82 (0.48)	−0.339 (−0.460 to −0.206)	** < 0.001**
QRS voltage (mV)	1.55 (0.71)	−0.531 (−0.626 to −0.419)	** < 0.001**

QRS voltage was calculated as the sum of the R voltage in V6 and the S voltage in V1.

Significant values are in bold.

## Discussion

In the present study, we demonstrated that a deep learning model, trained on a dataset consisting of low-weight individuals, achieved moderate accuracy (AUC, 0.807; 95% CI, 0.745–0.869) in predicting extremely low body weight, defined as BMI ≤ 12.6 kg/m^2^, using 12-lead ECG as input. Additional analyses highlighted the specific ECG features associated with the prediction.

Weight measurement in patients with AN is crucial owing to the risk of severe complications such as refeeding syndrome^24^, severe liver dysfunction^25^, and thrombocytopenia^25^. However, these patients tend to deny their disease status^19^ and may even falsify their weight by concealing heavy objects in their clothes during weigh-ins^20^. Furthermore, these patients may require intensive care^21^, where standard height and weight measurements may be difficult. Detecting extremely low body weight using a 12-lead ECG may be helpful in these cases. Such tools may become even more important given the reported increase in the prevalence of eating disorders since the onset of the COVID-19 pandemic^26^. However, deep learning models for ECGs are highly susceptible to even slight perturbations^27^, and defenses against such noise in deep learning have been widely investigated^28^. Future studies are required to assess the vulnerabilities of the models developed in the present study.

The predictive accuracy of the CNN model in the present study exceeded that of previous research aimed at predicting obesity (BMI > 25 kg/m^2^)^12,13^. These studies used datasets comprising > 10,000 individuals, possibly leading to considerable heterogeneity with wide ranges of comorbidities and ages. This heterogeneity may have complicated the prediction task. In contrast, our study used data predominantly from relatively young patients with AN showing monotonous physical comorbidity at a single institution, which may simplify the prediction task. To further evaluate the predictive ability of the deep learning model, validation using external datasets is required.

The Grad-CAM heatmap focused on the QRS waves, consistent with a strong correlation observed between a reduced QRS voltage and an elevated probability of extremely low body weight. These findings could be related to the frequent occurrence of pericardial effusions in patients with AN^29^, which can cause a low QRS voltage^30^. Although previous literature has indicated that QRS voltages are typically reduced in patients with AN^15^, the results of the present study suggest a quantitative relationship between greater weight loss and lower voltage, which constitutes a new finding.

Larger QT dispersion was positively, albeit insignificantly, associated with a higher likelihood of extremely low body weight. This might be related to previous findings that patients with AN exhibit greater QT dispersion than healthy controls^31^. In addition, although QTc prolongation is common in patients with AN^14,15^, its relationship with the CNN model prediction scores was insignificant. QTc prolongation is reportedly attributed to electrolyte disturbance rather than low body weight^32,33^, which may partially explain the lack of a significant relation.

The present study has several limitations. First, the sample size for deep learning development was relatively small. Enlarging the sample size or pre-training the model using an external dataset is desirable in future research. This limitation regarding the small sample size may have affected the model development process, such as the preprocessing methods. Second, the present study relied on data from usual clinical practice, in which ECG and weight measurements were not necessarily performed on the same day. This time lag may have influenced the results. Thus, future studies in which ECG and weight are measured simultaneously are warranted. Third, the limited availability of electronic medical records precluded us from obtaining detailed diagnostic categories such as restricting or binge-purging types. Fourth, in the present study, because the model inputs the waveform itself, available feature selection methods were limited. Future studies that can use more diverse feature selection methods are desirable. Finally, the present study used data collected at a single institution, potentially introducing some bias, such as data containing mostly patients with extremely severe AN under similar treatment. Further verification is required to determine whether the model developed in the present study can be applied to external data collected from multiple facilities.

In conclusion, using deep learning is feasible for predicting extremely low body weight based on 12-lead ECGs, which may be useful for specific cases, such as patients with AN attempting to falsify their weight. In addition, the model showed that several ECG features, such as lower QRS voltage, may be associated with extremely low body weight.

## Data Availability

The datasets analyzed during the current study are not publicly available because data sharing approval was not obtained from the institutional review board; however, they are available from the corresponding author on reasonable request.
